# Shrub Encroachment Rewires Microbial Networks to Suppress Soil Organic Carbon Mineralization in Subalpine Meadows

**DOI:** 10.1002/ece3.73922

**Published:** 2026-06-29

**Authors:** Pengli Hou, Jia Mi, Qianru Ren, Yao Su, Jincheng Shi, Jing Shi, Kuanhu Dong, Baofeng Chai

**Affiliations:** ^1^ Shanxi Key Laboratory for Ecological Restoration of Loess Plateau, Institute of Loess Plateau Shanxi University Taiyuan China; ^2^ Field Scientific Observation and Research Station of the Ministry of Education for Subalpine Grassland Ecosystem in Shanxi Ningwu China; ^3^ College of Environment and Resources Sciences Shanxi University Taiyuan China; ^4^ College of Grassland Science Shanxi Agricultural University Taigu China; ^5^ Observation and Research Station for Grassland Ecosystem in the Loess Plateau Youyu China

**Keywords:** carbon mineralization, microbial community, Q_10_, shrubification, subalpine meadow

## Abstract

Subalpine meadow ecosystems have undergone widespread shrubification over the past century due to climate change and anthropogenic activities, yet the impacts on soil organic carbon (SOC) mineralization and temperature sensitivity (Q_10_) remain unclear. We carried out laboratory incubation experiments with soils from native subalpine meadows and shrub‐encroached areas on Luliang Mountain. Soil properties, organic carbon fractions, and microbial communities were analyzed to assess their roles in SOC mineralization and Q_10_. The results showed that shrubification acidified soils, increased nutrients, reduced labile carbon (dissolved organic carbon, DOC), and elevated stable carbon (mineral‐associated organic carbon, MAOC). It raised microbial α‐diversity but reduced network complexity, with fungi responding more strongly. SOC mineralization rates decreased across depths due to lower pH, higher stable carbon, and disrupted microbial interactions. Total mineralization and Q_10_ in surface soil (0–5 cm) were notably reduced. Structural equation modeling indicated that in meadows, pH and nutrients co‐regulated mineralization, while under shrubification, nutrients dominated, altering microbial networks (weakening Pseudomonadota, Actinomycetota, Acidobacteriota, Ascomycota, and Basidiomycota interactions) to suppress SOC mineralization. These findings highlight that shrubification reshapes the key mechanisms of carbon mineralization, with soil nutrients playing a complex regulatory role in carbon dynamics. Our results from this typical subalpine region provide insights for predicting SOC mineralization feedbacks to climate change and understanding shrubification impacts on regional carbon balance.

## Graphical abstract

Shrub encroachment in subalpine meadows reduces soil organic carbon (SOC) mineralization rates and its temperature sensitivity (Q_10_), particularly in surface soil. This shift is driven by soil acidification, increased stable carbon content, and altered microbial network interactions. Our findings reveal that shrubification reshapes the regulatory mechanisms of carbon dynamics, with soil nutrients playing a dominant role in controlling mineralization under shrubs.

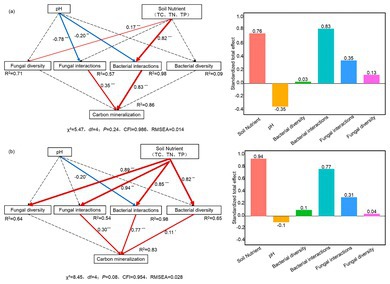

## 1 Introduction

Soil carbon storage represents the largest terrestrial carbon pool, exceeding the combined carbon stocks in global vegetation and the atmosphere (Wang et al. 2024). Alpine meadow ecosystems playing a critical role in regional and global carbon cycling, sequestering approximately 29% of soil organic carbon (SOC) due to low temperatures constraining microbial decomposition rate (Ding et al. 2019). However, the sensitivity of alpine ecosystems to climate change means that even minor alterations in soil carbon dynamics can significantly impact the global carbon balance. Over the past century, alpine meadows have undergone widespread shrub encroachment, termed meadow shrubification, driven by elevated CO_2_ concentrations, global warming, and anthropogenic disturbance (Chen et al. 2025). SOC mineralization, the microbial decomposition of organic matter into CO_2_, is central process governing soil carbon cycling (Cai et al. 2024), directly controlling SOC turnover, stability, and carbon emissions (Wang et al. 2024). Although alpine meadow shrubification is recognized to influence SOC mineralization, its specific impact on CO_2_ emissions via temperature‐sensitive mineralization remains poorly understood.

Shrubification remodels shrub‐herb competition to restructure plant community and biodiversity and redistributes soil water, nutrients, and microorganisms to form fertile islands (Zhang et al. 2026); resource competition increases shrub coverage and biomass alongside the degradation of native herbs, and positive feedback between altered vegetation and soil heterogeneity accelerates shrubification and soil nutrient loss (Hou et al. 2025), while the fertile‐island effect improves local soil resource conditions and productivity, ultimately leading to divergent positive and negative regulatory effects of shrubification on vegetation‐soil systems (Song et al. 2026). Shrubification alters soil physicochemistry, carbon fractions, and microbial communities, thereby influencing ecosystem function (Chen et al. 2023). Root secretions and litter inputs from shrubs modify soil nutrient supply (Gao et al. 2024) and microenvironments (moisture, nutrients, pH), which in turn reshape microbial community structure (Zhang et al. 2022). Changes in litter quality and root exudates during shrubification also affect microbial community composition, diversity, and network complexity (Xiang et al. 2018). Microbial networks enhance community stability and functional redundancy through interspecific interactions, profoundly influencing SOC mineralization (Wan et al. 2023). Shrubification disrupts the original community balance, altering microbial biomass and enzyme activities (Wang, Bian, et al. 2021), though its effects on alpine meadow carbon decomposition remain uncharacterized.

Temperature regulates SOC mineralization by directly influencing microbial activity and indirectly altering soil physicochemistry (Huang et al. 2019). Temperature changes affect soil porosity and moisture, modulating carbon mineralization rates (Bonfanti et al. 2025). Warmer temperatures typically enhance microbial metabolism, accelerating organic carbon decomposition (Li et al. 2024). For example, temperature sensitivity (Q_10_) values, indicators of temperature sensitivity, decrease with increasing temperature across ecosystems, suggesting weakened temperature sensitivity of SOC mineralization under heat stress (Wang, Bian, et al. 2021; Wang, Morrissey, et al. 2021). Elevated temperatures also accelerate SOC decomposition, providing more labile carbon that influences microbial community structure (Wang et al. 2025), while promoting nutrient release to feedback on carbon‐nitrogen cycling (Gudasz et al. 2010). Current research indicates that the mineralization of global SOC results in the release of approximately 57 to 80 Pg C to the atmosphere as CO_2_ each year, which is nearly 9% of the atmospheric carbon pool. Climate warming may accelerate SOC mineralization, leading to positive soil carbon cycle‐climate change feedbacks (Tang et al. 2020). Temperature modulated shrubification, an increasingly observed phenomenon in alpine and high, latitude ecosystems can significantly alter soil microenvironments and carbon accumulation patterns (Qiao et al. 2023). However, the specific effects of temperature on SOC mineralization under shrubification conditions remain largely unexamined, especially across diverse climatic zones and soil types. Although Q_10_ plays a crucial role in modeling SOC dynamics under global warming, the mechanisms by which shrubification influences SOC mineralization and its Q_10_ are still not well understood. This represents a critical knowledge gap that hinders accurate predictions of carbon‐climate feedbacks in shrubification of subalpine meadows under future climate scenarios.

The Luliang Mountains, featuring typical North China subalpine meadows, are undergoing rapid shrubification. This study aimed to quantify how shrubification alters SOC mineralization and its Q_10_ along soil vertical profiles, and further unravel the underlying microbial regulatory mechanisms. We compared soil physicochemical properties, organic carbon fractions, and microbial community compositions between intact subalpine meadows and shrubification sites, coupled with a 63‐day laboratory aerobic incubation experiment. We hypothesized that shrubification would: (1) modify soil properties and disrupt microbial symbiosis networks; (2) reduce SOC mineralization rates and Q_10_ values, indicating weakened temperature sensitivity; (3) regulate mineralization through carbon fraction mediated restructuring of microbial communities and functionality.

## 2 Materials and Methods

### 2.1 Study Area

The experiment was conducted in Shanxi Luya Mountain National Nature Reserve. The experimental site is located in Heyeping of Luliang Mountain (38°40′–38°50′ N, 111°50′–112°00′ E), at an altitude of 2784 m. The Luliang Mountains is situated has a series of Tibetan flora in subalpine meadow, which makes this area's mountains highly representative in terms of natural history and plants. Moreover, the subalpine meadows of the Luliang Mountains are mainly distributed in the Luya Mountain area. The region has a warm‐temperate continental climate with cool, rainy summers and cold, dry winters, featuring an average annual temperature of 4°C–7°C, 10°C–20°C during the growing season, 384–679 mm annual precipitation, and 1800 mm annual evapotranspiration (concentrated in May–October). Two sampling sites (2 km apart, similar altitude/climate) were selected: native subalpine meadows dominated by herbaceous plants *Polygonum viviparum, Kobresia pygmaea, Carex appropinquata* with companions *Thalictrum squarrosum, Carex montis‐wutaii, Elymus dahuricus, Potentilla fragarioides*, and shrubification of subalpine meadows dominated by *Caragana jubata* with companions *Artemisia* spp., *Pulsatilla chinensis, Bidens* spp., *Caragana* spp., *Lespedeza* spp.

### 2.2 Sample Collection

In late September 2024, three 1 × 1 m plots were established per site. After removing aboveground biomass, soil samples were collected from 0–5, 5–10, and 10–20 cm depths using a 5 cm diameter soil coring drill (double diagonal five‐point sampling per plot). A total of 18 composite samples were obtained by mixing five soil cores per plot across two site types (native subalpine meadows and shrubification of subalpine meadows), with three replicate plots per type and three soil depths per plot. The soil samples were rapidly transported back to the laboratory. In the laboratory, plant roots, gravel, and other debris were first removed from the soil samples, which were then sieved through a 2 mm sieve and thoroughly mixed. The soil was then divided into two portions. One portion was immediately placed into insulated coolers with ice packs during field transportation and stored at −20°C in the laboratory for approximately 2 months prior to microbial analyses. The remaining soil was air‐dried under cool ambient conditions for physicochemical analysis and incubation experiments.

### 2.3 SOC Mineralization Experiment

Soil depths (0–5, 5–10, and 10–20 cm) were incubated at 15°C, 25°C, and 35°C for 63 days in a dark incubator. The experiment comprised 54 units: two meadow types × three depths × three temperatures × three replicates. The incubation was conducted at 60% of field water holding capacity (WHC). Deionized water was added during the incubation period, and the moisture content of all samples was adjusted to 60% of WHC using the gravimetric method. Incubation bottles were covered with perforated plastic wrap to facilitate gas exchange and minimize evaporative water loss. Gravimetric weighing for water loss was conducted every 2 days at 20:00; deionized water was pipetted dropwise onto the soil surface to restore moisture to 60% WHC, and water was allowed to infiltrate slowly into the soil.

Twenty grams of each soil sample were weighed and placed into 110 mL incubation flasks (inner diameter 3 cm). To homogenize moisture distribution and restore soil microbial activity, samples were pre‐incubated in a 25°C constant‐temperature incubator for 7 days. After pre‐incubation, samples were transferred to incubators set at 15°C, 25°C, and 35°C for main incubation, with flasks without soil used as controls. Gas sampling was performed on Days 1, 2, 3, 7, 14, 21, 28, 35, 42, 49, 56, and 63 of the main incubation. Prior to each sampling, flasks were sealed with rubber stoppers, and two syringes connected to an air pump were used to flush the headspace with air for 5 min. After flushing, flasks were resealed and returned to the incubators. Two hours later, 10 mL gas samples were extracted using syringes with three‐way valves and immediately analyzed for CO_2_ concentration using a gas chromatograph (GC Agilent 7820).

CO_2_ concentrations were converted to CO_2_‐C using the ideal gas law (Robertson et al. 1999):

(1) $$C_{m}=\frac{\left(C_{v}\times M\times P\right)}{\left(R\times T\right)}$$

(2) $$F=\frac{C_{\text{rate}}\times V}{W}$$

where *C*_*m*_ (μg CO_2_‐C L^−1^) is the concentration change, *C*_*v*_ (ppm) is CO_2_ volume, *M* = 12 μg/μmol, *P* is atmospheric pressure, *R* = 0.0820575 L·atm·K^−1^·mol^−1^, *T* (K) is temperature, *V* (L) is headspace volume, *W* (g) is dry soil weight. *C*_*rate*_ represents the change in CO_2_ concentration within the incubation flask during soil incubation, calculated from *C*_*m*_ (μg CO_2_‐C L^−1^ d^−1^). *F* denotes the carbon mineralization rate (mg CO_2_‐C kg^−1^ soil d^−1^).

Cumulative mineralization was calculated as follows:

(3) $$C=\sum_{i=1}^{n}\frac{\left(F_{i}+F_{i+1}\right)}{2\times \left(t_{i+1}-t_{i}\right)}\times 24$$

where *C* represents the cumulative SOC mineralization (mg kg^−1^), and *F* denotes the SOC mineralization rate (mg kg^−1^ h^−1^). Here, *i* is the measurement number, *tᵢ*_+1_‐*tᵢ* is the time interval between two consecutive measurements, and *n* is the total number of measurements during the incubation period.

The cumulative SOC mineralization refers to the total CO_2_ release (as CO_2_‐C) from the start of incubation to a specific time point. The dynamic change in cumulative SOC mineralization with incubation days was modeled using the first‐order kinetic equation with R (Stanford and Smith 1972):

(4) $$f\left(x\right)=C_{0}\times \left(1-e^{-\mathrm{kx}}\right)$$

where *C*_*t*_: Cumulative mineralization of SOC after time *t* (g/kg); *C*_0_: Potential mineralizable organic carbon in soil (g/kg); *k*: Turnover rate constant of the organic carbon pool (d^−1^); *t*: Incubation days (d).

The half‐turnover period ($T_{1/2}$) is calculated as:

(5) $$T_{1/2}=\ln\frac{2}{k}$$

The Q_10_ is calculated as follows:

(6) $$Q_{10}={\left(\frac{F_{\text{high}}}{F_{\mathrm{low}}}\right)}^{\frac{10}{\Delta T}}$$

where *F*_*high*_ and *F*_*low*_: Total cumulative SOC mineralization under high and low temperature treatments, respectively; Δ*T*: Temperature difference between high and low treatments (Δ*T = T*_*high*_−*T*_*low*_). We calculated Q_10_ values from 15°C to 25°C and from 25°C to 35°C for comparison.

### 2.4 Soil Physicochemical Analysis

Soil pH was measured at 1:5 soil: water using a glass electrode. WHC was determined by weighing. Total C (TC), N (TN), and SOC were analyzed via dry combustion (Vario Micro Cube). Total P (TP) was measured by molybdenum blue spectrophotometry (Shimadzu UV‐1800). Microbial biomass carbon (MBC) was quantified by chloroform‐fumigation K_2_SO_4_ extraction; dissolved organic carbon (DOC) was extracted from unfumigated extracts. Particulate organic carbon (POC) and mineral‐associated organic carbon (MAOC) were separated by 0.5% sodium hexametaphosphate dispersion, sieving (0.053 mm), and analyzed per Shi, Delgado‐Baquerizo, et al. (2024) and Shi, Deng, et al. (2024).

### 2.5 DNA Extraction and Sequencing

High‐throughput sequencing was performed to assess bacterial and fungal community composition and diversity, using primers 338F (5′‐ACTCCTACGGGAGGCAGCA‐3′) and 806F (5′‐GGACTACHVGGGTWTCTAAT‐3′) for 16S rRNA genes, and primers ITS1F (5′‐CTTGGTCATTTAGAGGAAGTAA‐3′) and ITS2R (5′‐GCTGCGTTCTTCATCGATGC‐3′) for ITS genes. Amplification products were sequenced on an Illumina MiSeq platform (Majorbio Biotechnology Co. Ltd., Shanghai, China).

### 2.6 Statistical Analysis

Two‐way ANOVA was used to analyze the effects of vegetation type, soil depth, and their interaction on soil nutrients, carbon fractions, microbial diversity, and community structure. Three‐way ANOVA was applied to examine the effects of vegetation type, soil depth, and incubation temperature (as three fixed factors) on SOC mineralization after the completion of incubation. Pearson correlation analysis was used to explore the relationships between carbon fractions, microbial communities, diversity, stoichiometric ratios, and organic carbon mineralization (*p <* 0.05). Nonmetric multidimensional scaling (NMDS) was performed to analyze the composition of microbial community structures in meadows at different depths. Based on ASV data, node and edge attributes were extracted using the “igraph” package in R 4.4.2 to calculate network parameters such as the number of nodes, average degree, network diameter, and average path length. Microbial ecological networks were visualized using Gephi V0.9.2, and co‐occurrence networks were used to analyze interactions between microbial species. Combined with the “igraph” and “ggplot2” packages, the *p*‐values of species association matrices were calculated, node topological positions were analyzed, key species were identified, and the co‐occurrence patterns of microbial communities and the role of core taxa were clarified. To explore the mechanism by which shrubification affects microbially mediated SOC mineralization, this study constructed a SEM using AMOS software to analyze the causal relationships between soil nutrients, bacterial and fungal diversity, microbial topological parameters, and soil organic carbon mineralization. First, principal component analysis (PCA) was performed on soil nutrients (TC, TN, TP), microbial diversity (Sobs, ACE, Chao 1, Shannon, Simpson, Shannoneven, Simpsoneven, Coverage), and network topological parameters (Edge, Node, Proportion of positive edges (%), Average degree, Weighted degree, Modularity, Average clustering coefficient), as well as carbon mineralization rates (cumulative carbon mineralization at 15°C, 25°C, and 35°C) to reduce collinearity. The first principal component (PC1) from the PCA analysis was introduced into the model, with PC1 explaining over 50% of the variance in each group, indicating that using PC1 as a new variable in SEM to explain the overall variation of each group of variables is reliable (Table S1). The SEM was constructed based on the dimensionality reduction results. Finally, multiple diagnostic tests (*χ* ^2^, df, RMSEA, CFI, etc.) were used to verify the goodness‐of‐fit of the SEM model, quantifying the direct and indirect effects of shrubification on SOC mineralization, and clarifying the mechanism by which soil properties and microbial characteristics synergistically drive carbon mineralization. The main R packages used include: “igraph” for constructing and visualizing microbial co‐occurrence networks, and “ggplot2” for data visualization. All statistical analyses were performed using Microsoft Excel 2019, IBM SPSS 27.0, and R 4.4.2. Prior to statistical analysis, all data were tested for normality and homogeneity of variance. *p <* 0.05 was considered statistically significant for differences between means.

## 3 Results

### 3.1 Soil Physicochemical Properties and Carbon Fractions

**FIGURE 1:**
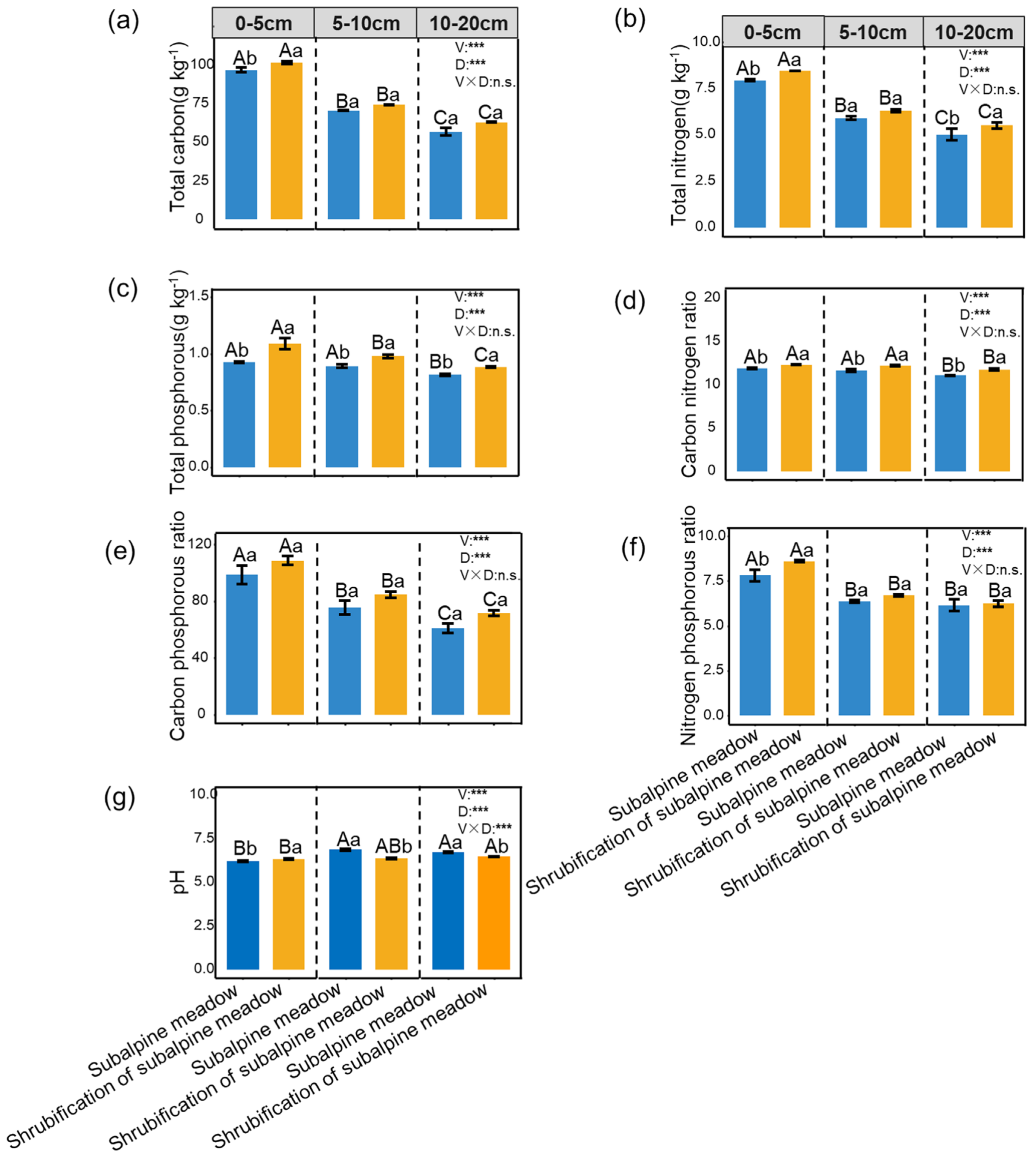
TC (a), TN (b), TP (c) contents, soil C/N (d), C/P (e) and N/P (f) ratios, and soil pH (g) values in 0–5 cm, 5–10 cm, and 10–20 cm soil depths of native subalpine meadow and shrubification of subalpine meadow. Error bars represent standard deviations. V, vegetation type; D, soil depth; V × D, the interaction effect between vegetation type and soil depth. Different uppercase letters indicate significant differences in means between different soil depths under the same vegetation type. Different lowercase letters indicate significant differences in means between different vegetation types within the same soil depth. ANOVA results are also provided: ***p < 0.001, n.s. not significant.

Two‐way ANOVA showed that soil pH differed significantly (*p <* 0.001) between vegetation types, soil depths, and the interaction between vegetation types and soil depths (Figure 1). Compared with meadows, shrubification significantly reduced soil pH at soil depths of 0–5, 5–10, and 10–20 cm (*p <* 0.05) (Figure 1g). TC, TN, TP, C/N, C/P, and N/P values differed significantly (*p <* 0.05; Figure 1a–f) in terms of vegetation type and soil depth as a single factor, but not in terms of the interaction between vegetation type and soil depth (*p* > 0.05). Concentrations of TN, TC, and TP were significantly increased by shrubification‐induced nutrient enrichment, and TN, TC, and TP were significantly higher (*p <* 0.05) in shrub‐encroached subalpine meadows than in native subalpine meadows. Similarly, soil C/N, C/P, and N/P were all significantly higher (*p <* 0.05) in shrubification of subalpine meadows compared to meadows. On the soil depth gradient, soil TC, TN, and TP contents and their ratios were higher (*p <* 0.05) from 0–5 cm than from 5–10 cm and from 10–20 cm.

**FIGURE 2:**
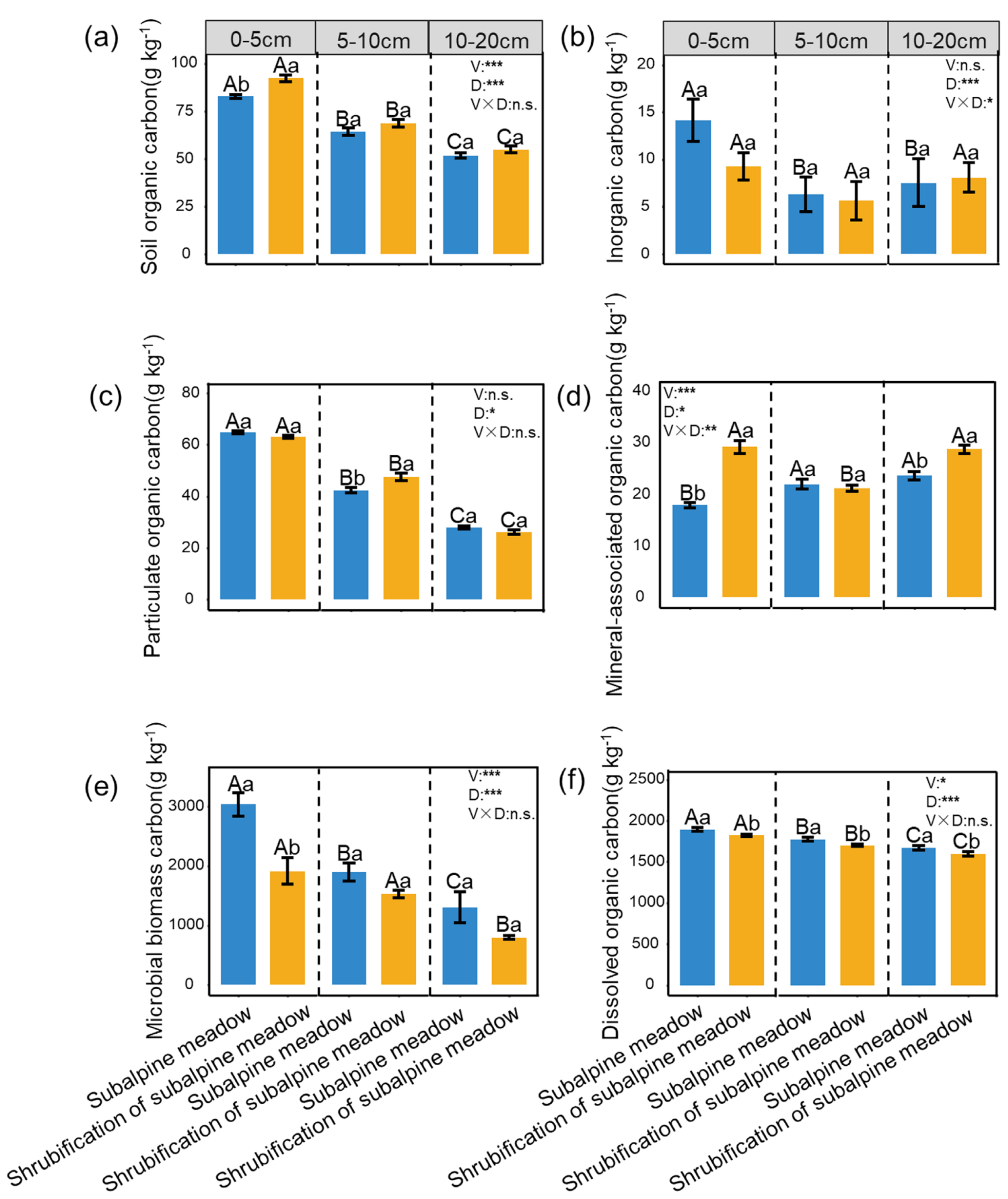
SOC (a), IC (b), POC (c), MAOC (d), MBC (e) and DOC (f) contents in 0–5 cm, 5–10 cm, and 10–20 cm soil depths of native subalpine meadow and shrubification of subalpine meadow. Error bars represent standard deviations. V, vegetation type; D, soil depth; V × D, the interaction effect between vegetation type and soil depth. Different uppercase letters indicate significant differences in means between different soil depths under the same vegetation type. Different lowercase letters indicate significant differences in means between different vegetation types within the same soil depth. ANOVA results are also provided: ***p < 0.001, **p < 0.01, *p < 0.05, n.s. not significant.

Two‐way ANOVA showed that soil DOC, SOC, MBC, and POC differed significantly (*p <* 0.001) with respect to vegetation type and soil depth (Figure 2a,c,e,f), but not with respect to the interaction between vegetation type and soil depth (*p* > 0.05). Compared with meadows, shrubification significantly reduced DOC values at 0–5, 5–10, and 10–20 cm soil depth (*p <* 0.05); MBC values at 0–5 cm soil depth; POC values at 0–5 and 5–10 cm soil depth; and significantly increased SOC values at 0–5 cm soil depth (*p <* 0.05; Figure 2); and IC content was higher than that of shrubification (Figure 2b); In contrast, soil MAOC values differed significantly with vegetation type and soil depth, and a significant interaction between vegetation type and soil depth was also detected (*p* < 0.05; Figure 2d). Compared with meadows, shrubification of subalpine significantly increased soil MAOC values in the 0–5 cm and 10–20 cm soil horizons (*p <* 0.05).

### 3.2 The Temperature Sensitivity of SOC Mineralization

**FIGURE 3:**
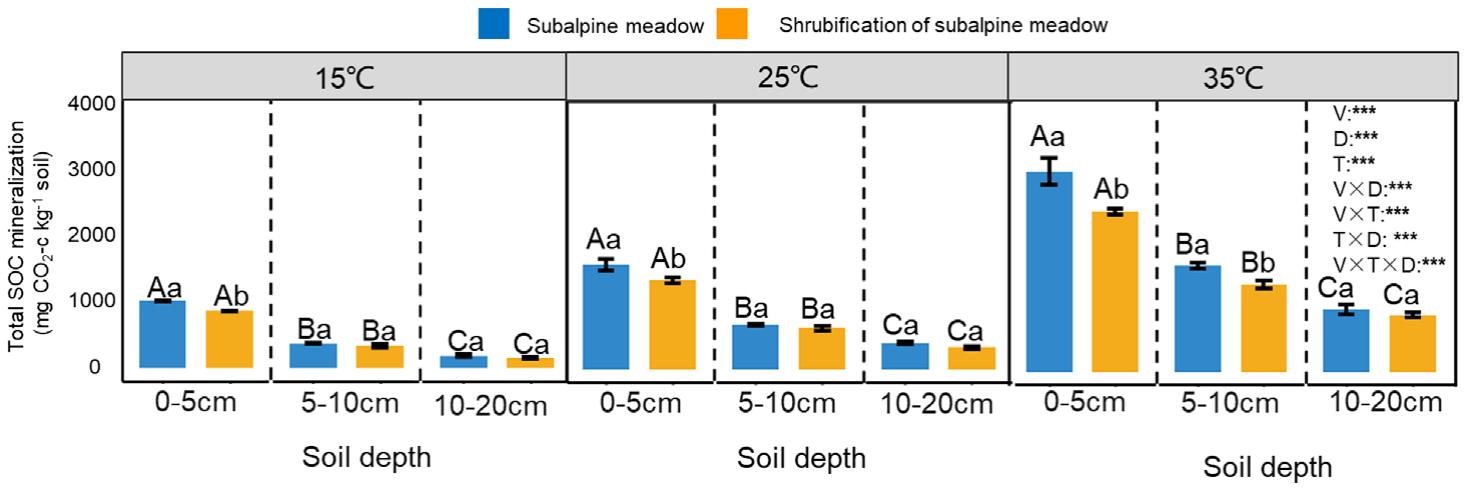
Effects of soil depth (0–5 cm, 5–10 cm, and 10–20 cm) and incubation temperature (15°C, 25°C, and 35°C) on total SOC mineralization (evolved as CO_2_, mg C g^−1^ SOC) over 63 days in native subalpine meadow and shrubification of subalpine meadow. Error bars represent standard deviations. V, vegetation type; D, soil depth; T, temperature, V × D, the interaction effect between vegetation type and soil depth. V × T, the interaction effect between vegetation type and temperature, T × D, the interaction effect between temperature and soil depth. V × T × D, the three‐way interaction effect between vegetation type, temperature, and soil depth. Different uppercase letters indicate significant differences in means between different soil depths under the same vegetation type. Different lowercase letters indicate significant differences in means between different vegetation types within the same soil depth. ANOVA results are also provided: ***p < 0.001.

Overall, throughout the entire incubation period at 15°C, 25°C, and 35°C, the shrubification of subalpine meadow reduced the soil SOC mineralization rate compared to the native subalpine meadow. At the end of the 63‐day incubation, the shrubification of subalpine meadow decreased the total SOC mineralization (total carbon converted to CO_2_ per unit mass of SOC) across all soil depths relative to the native subalpine meadow: in the 0–5 cm depth, the total SOC mineralization in the shrubification of subalpine meadow was reduced by 20.1%; in the 5–10 cm and 10–20 cm depths, the total SOC mineralization in the shrubification of subalpine meadow decreased by 17.5% and 12.2%, respectively (Figure 3). In terms of differentiating factors, soil organic carbon mineralization in both the native subalpine meadow and the shrubification of subalpine meadow was interactively influenced by temperature, soil depth, and ecological type: with increasing temperature, carbon mineralization significantly increased across all soil depths; with increasing soil depth, carbon mineralization significantly decreased. Regarding ecological type, the difference in carbon mineralization between the shrubification of subalpine meadow and the native subalpine meadow was regulated by the interaction of temperature and soil depth: at 15°C, the differences among soil depths were small; at 25°C, the shrubification of subalpine meadow showed a slight increase in carbon mineralization in the 0–5 cm depth; at 35°C, carbon mineralization in the 0–5 cm depth of the shrubification of subalpine meadow was significantly lower than that of the native subalpine meadow, while the differences in deeper depths narrowed.

**FIGURE 4:**
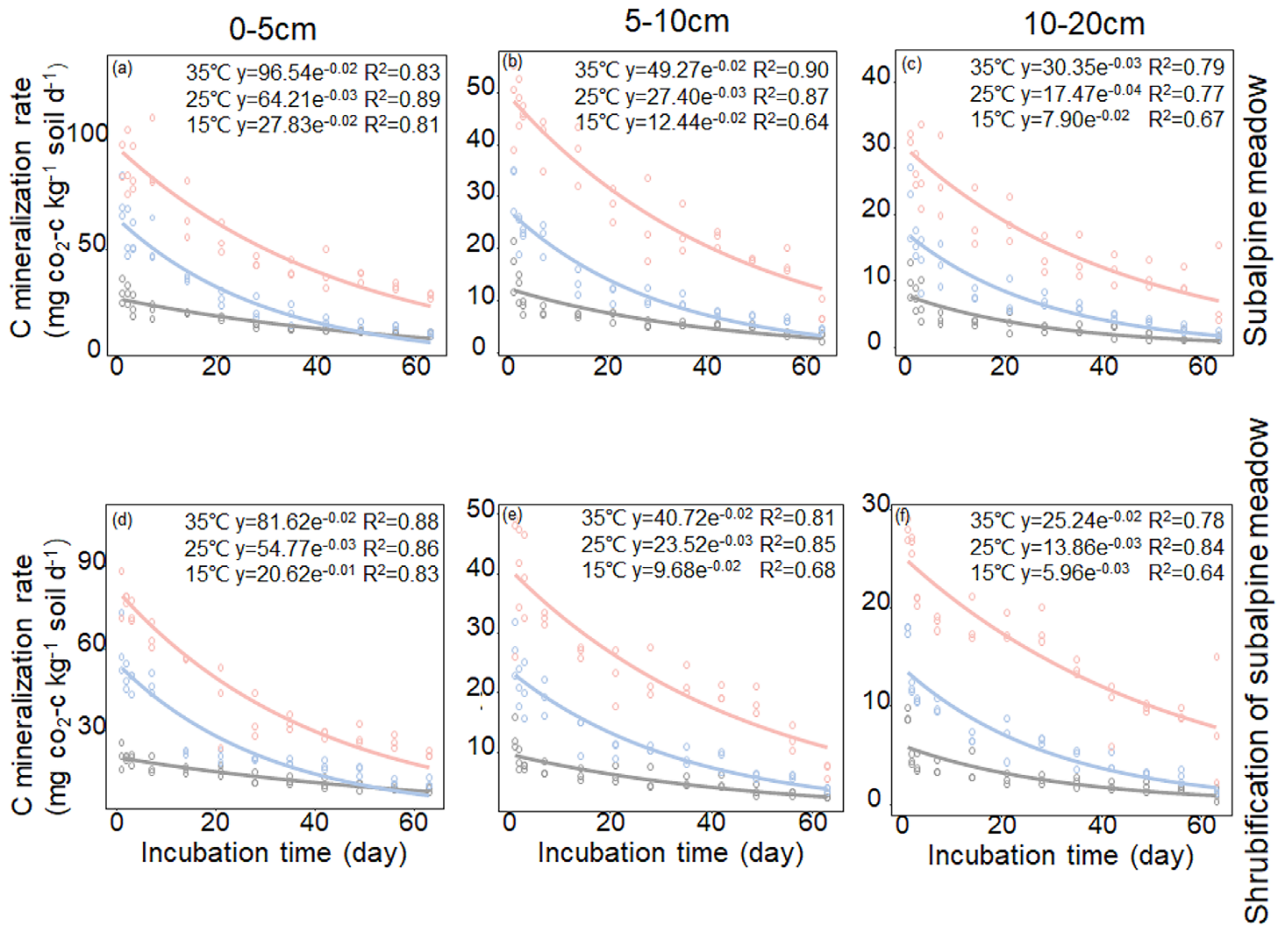
Decay dynamics of soil CO_2_ mineralization in 0–5 cm, 5–10 cm, and 10–20 cm soil depths of native subalpine meadow and shrubification of subalpine meadow under incubation temperatures (15°C, 25°C, and 35°C). All fitting results reached a significant level (*p <* 0.05).

Soil CO_2_ mineralization rates showed significant variations with temperature, soil depth, and vegetation type over the 9‐week incubation period (Figure 4). Overall, all soil carbon emissions decreased exponentially with time. The dynamics of carbon mineralization rate can be accurately described by an exponential decay function with two parameters (Formula: 2–4) (*p <* 0.001). The initial mineralization rate (*C*_*0*_ value) estimated from the exponential decay function increased with increasing incubation temperature within each treatment. At the same incubation temperature, the highest initial mineralization rates were usually observed in 0–5 cm, followed by 5–10 cm, and finally 10–20 cm. Comparing the native subalpine meadow soils to the scrubby soils, the initial mineralization rates were significantly higher in the native subalpine meadow soils than in the shrubification soils. In contrast, decay constants (*k* values) showed different patterns, being higher at 25°C and lower at 15°C and 35°C.

**FIGURE 5:**
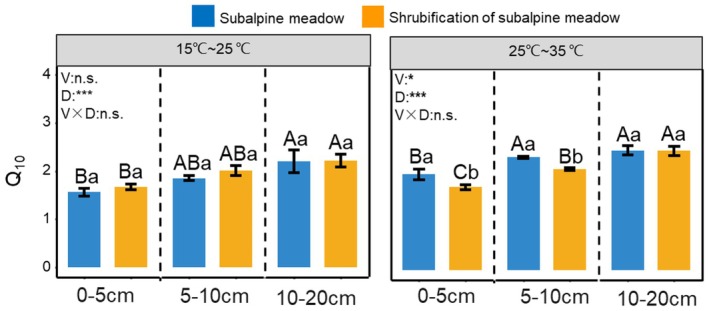
Effects of soil depth (0–5 cm, 5–10 cm, and 10–20 cm) and temperature range (15°C–25°C and 25°C–35°C) on the total Q_10_ values over 63 days in native subalpine meadow and shrubification of subalpine meadow. Error bars represent standard deviations. V, vegetation type; D, soil depth; V × D, the interaction effect between vegetation type and soil depth. Different uppercase letters indicate significant differences in means between different soil depths under the same vegetation type. Different lowercase letters indicate significant differences in means between different vegetation types within the same soil depth. ANOVA results are also provided: ***p < 0.001, *p < 0.05, n.s. not significant.

Between 15°C and 25°C, Q_10_ values differed significantly (*p <* 0.05) with respect to soil depth (Figure 5), but not with respect to the interaction of vegetation type, vegetation type and soil depth (*p* > 0.05), and there were no significant differences in Q_10_ values between the soil depths of the native subalpine meadow and the shrubification of subalpine meadows. Between 25°C and 35°C, Q_10_ values differed significantly (*p <* 0.05) in terms of vegetation type and soil depth, but not in terms of the interaction between vegetation type and soil depth (*p* > 0.05), with shrubification significantly lowering Q_10_ values in the 0–5 cm and 5–10 cm soil layers at the 25°C–35°C temperature range.

### 3.3 Effects of Shrubification on Soil Bacterial and Fungal Community Composition, Diversity, and Symbiotic Patterns in Shrubification of Subalpine Meadows

The bacterial community primarily comprises Pseudomonadota, Acidobacteriota, Actinomycetota, Bacteroidota, and Chloroflexota. Ascomycota, Mortierellomycota, and Basidiomycota are the dominant phyla in the fungal community (Figure S1). NMDS analysis revealed consistent results for both bacterial and fungal communities, with significant differences in the distribution of microbial community structures between shrubification and subalpine meadows (*p* < 0.05, Figure S3). Compared with bacteria, the community differences (*F*‐value) among fungi were more pronounced. Additionally, the β‐diversity of these microbial communities showed significant changes (*p* < 0.01, Figure S3). Fungal diversity and richness also differed significantly between different meadows (Figure S2). Indices of richness (Sobs, Chao), diversity (Shannon, Simpson, Shannoneven, Simpsoneven), richness estimation (ACE), and coverage (Coverage) for both bacterial and fungal communities exhibited significant differences under the individual effects of vegetation type and soil depth (*p* < 0.05). Fungal communities differed significantly between shrubification of subalpine meadows and native subalpine meadows (*p* < 0.05). The interaction of vegetation type and soil depth had significant effects on some indices of bacterial communities (Shannon, Simpsoneven) and fungal communities (Sobs, Chao, Shannon) (*p* < 0.05). Significant differences were observed in microbial communities (fungi and bacteria in the 0–5 cm depth) between native subalpine meadows and shrubification of subalpine meadows across all soil depths (*p* < 0.05), with increased α‐diversity of microbial communities in shrubification of subalpine meadows (*p* < 0.05).

**FIGURE 6:**
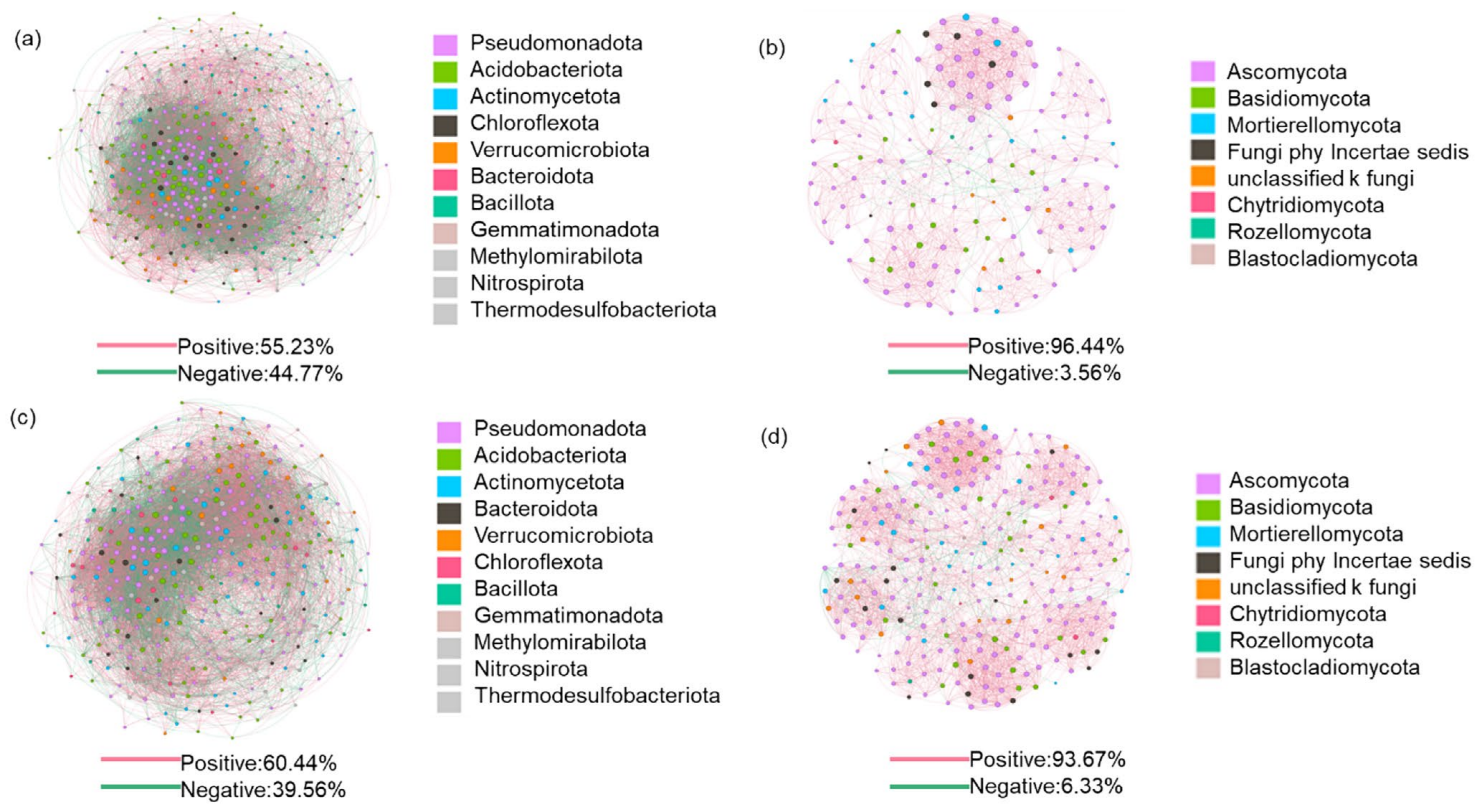
Co‐occurrence networks of phylum‐level bacterial (a) and fungal (b) communities in native subalpine meadow, phylum‐level bacterial (c) and fungal (d) communities in shrubification of subalpine meadow. Each node in the network corresponds to a phylum. Nodes are color‐coded to distinguish microbial taxa, with nodes of the same color belonging to the same phylum. Nodes represent microbial taxa, and edges represent co‐occurrence relationships between species, where pink edges indicate positive correlations and green edges indicate negative correlations.

**FIGURE 7:**
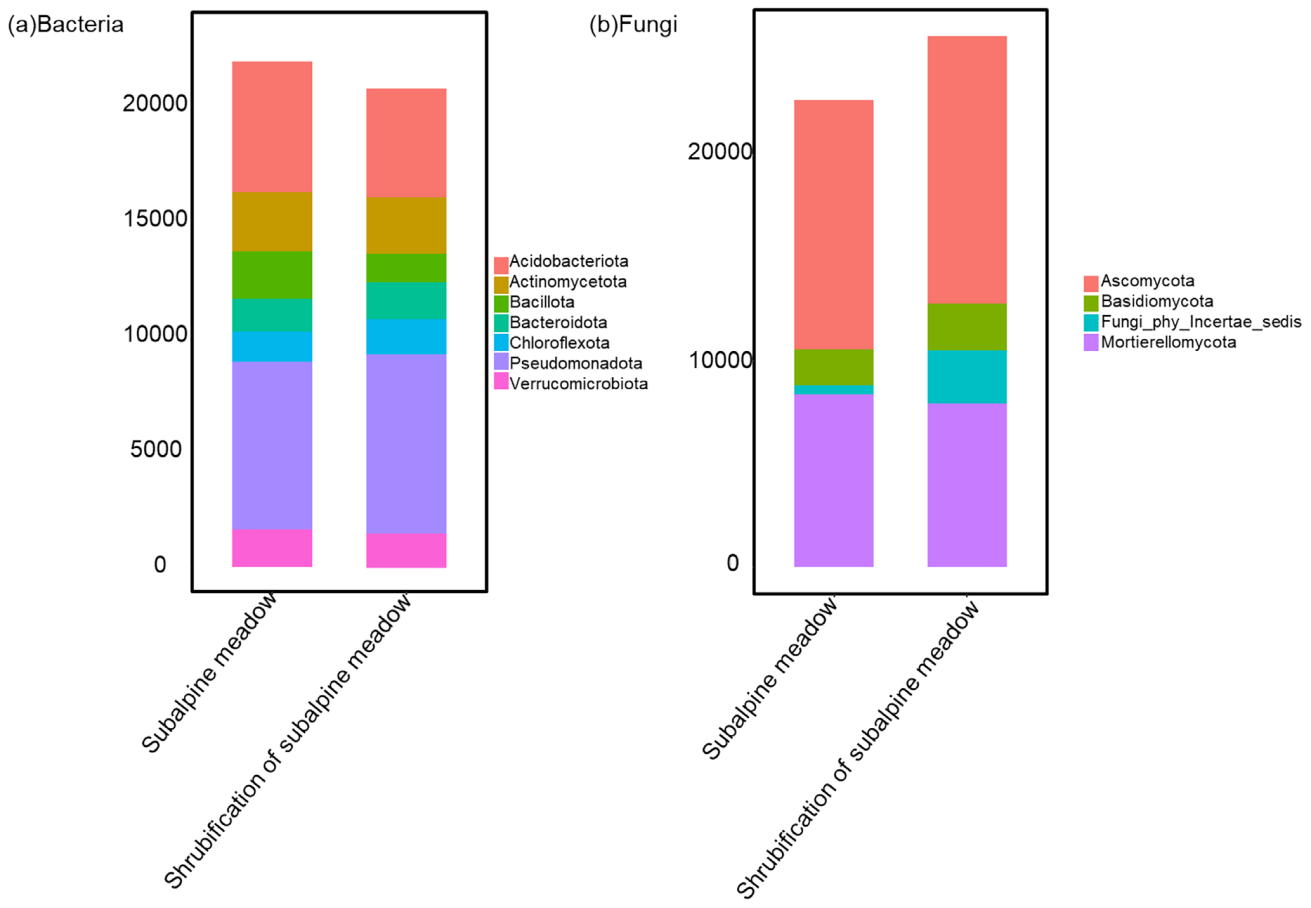
The composition of key species in the microbial communities of native subalpine meadows and shrubification of subalpine meadows.

Co‐occurrence networks were constructed to identify distinct symbiotic patterns of soil bacterial and fungal communities (Figure 6). The fungal and bacterial communities in meadows included 11 and 8 phyla in their respective co‐occurrence networks, and the topological properties of microbial co‐occurrence networks reflected the connectivity and interaction intensity among microbial communities. Shrubification significantly altered the interaction patterns of bacterial and fungal communities in meadows. In the studied meadows, the most critical phylum in the bacterial community was Pseudomonadota, followed by Acidobacteriota and Actinomycetota. The dominant phylum in the fungal community was Ascomycota, followed by Basidiomycota and Mortierellomycota. Based on the network diagrams, the key species composition of microbial communities in different meadows was identified (Figure 7). Most key species in the meadow network belonged to Pseudomonadota, Bacillota, Bacteroidota, Chloroflexota, Verrucomicrobiota, Actinomycetota, and Acidobacteriota (Figure 6a), while the key species of fungal communities in native subalpine meadows were mainly Ascomycota, Mortierellomycota, and Basidiomycota (Figure 6b).

The topological parameters of microbial communities are shown in Table 1. Compared with native subalpine meadows, positive correlations were more prevalent than negative correlations in bacterial networks of shrubification of subalpine meadows; however, positive correlations in fungal networks were lower. The number of edges, nodes, average degree, and weighted degree of bacterial networks in native subalpine meadows were 1.46, 1.05, 1.39, and 1.50 times those in shrubification of subalpine meadows, respectively, indicating that bacterial networks in native subalpine meadows were more extensive, more tightly connected, and more structurally complex. The average degree, graph density, and average clustering coefficient reflect the connectivity and aggregation degree of nodes in the network. Smaller average path length and network diameter indicate higher efficiency of information transmission and material exchange among species. Compared with bacterial networks in native subalpine meadows, those in shrubification of subalpine meadows showed lower values in clustering coefficient (−13.2%), average path length (2.74%), and modularity (30.3%), suggesting that native subalpine meadows respond more rapidly to external conditions. The number of edges, nodes, average degree, and weighted degree of fungal networks in native subalpine meadows were 0.44, 0.62, 0.71, and 0.72 times those in shrubification of subalpine meadows, respectively. Compared with fungal networks in native subalpine meadows, those in shrubification of subalpine meadows exhibited lower clustering coefficient (−8.70%), diameter (−40%), and average path length (−9.86%), indicating higher energy transfer efficiency of fungal communities in native subalpine meadows. The modularity values of fungal networks in meadows were all greater than 0.40, suggesting that microbial networks have good aggregation effects and close connections among modules.

**TABLE 1:** Network topological properties of microbial interactions at the phylum classification level.

Meadow type	Microorganism	Edge	Node	Proportion of positive edges (%)	Average degree	Weighted degree	Modularity	Average clustering coefficient
Subalpine meadow	Bacteria	14,985	392	55.23	76.45	82.65	0.23	0.60
	Fungi	1040	158	96.44	16.92	21.69	0.74	0.75
Shrubification of subalpine meadow	Bacteria	10,297	375	60.44	54.92	55.10	0.33	0.53
	Fungi	3177	266	93.67	23.89	25.52	0.76	0.69

### 3.4 Influence of Soil Properties, Microbial Communities and Carbon Fractions on SOC Mineralization

**FIGURE 8:**
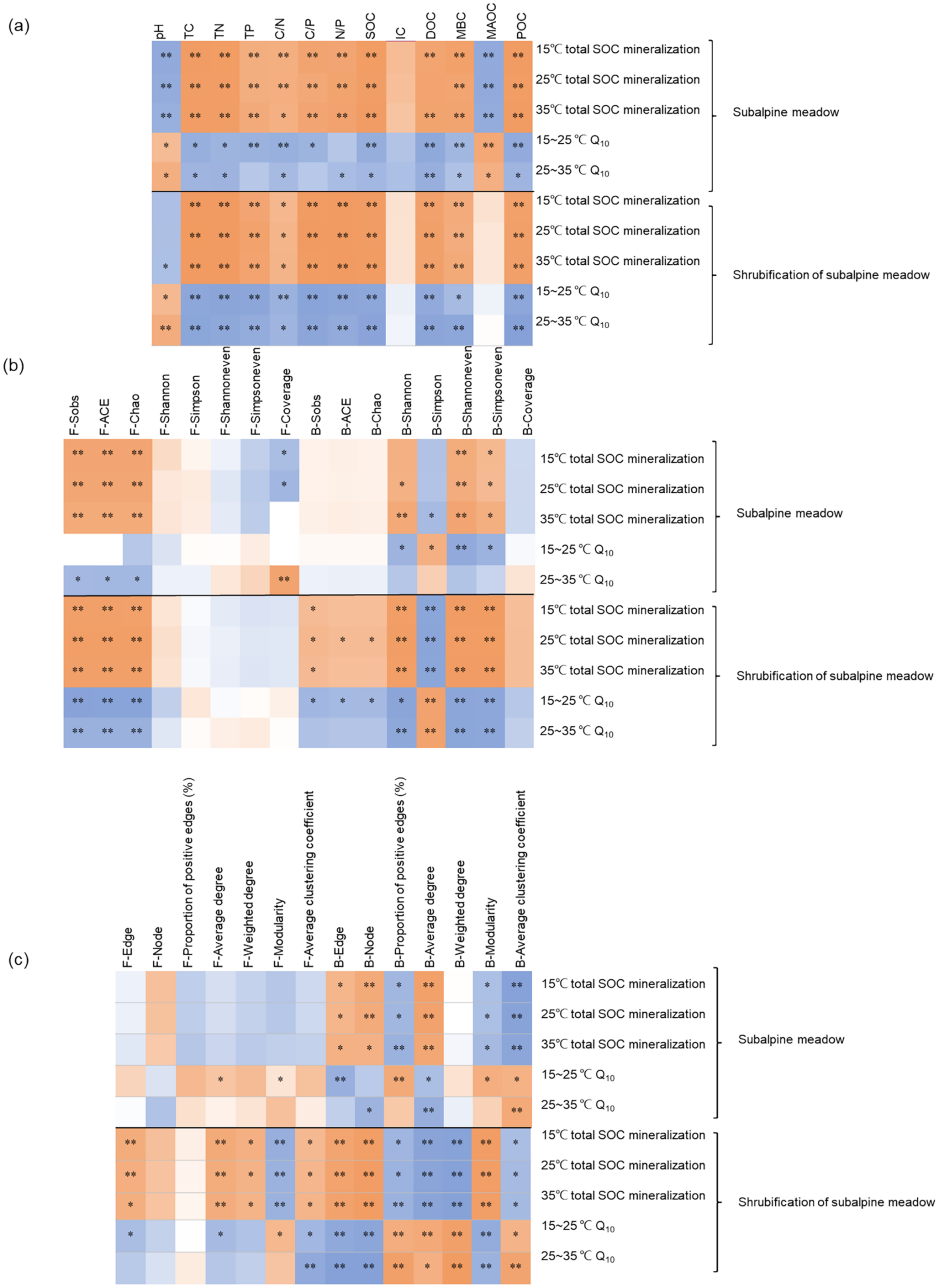
Correlations among mineralization, physicochemical properties (a), microbial diversity indices (b), and network topological logical characteristics (c). The color gradient in the heatmap represents the Pearson correlation coefficient. Orange indicates a positive correlation, while blue indicates a negative correlation. Asterisks denote the significance levels (**p < 0.01, *p < 0.05).

SOC mineralization is influenced by multiple factors in the soil microenvironment (Figure 8). Our results showed that there were significant relationships between cumulative mineralization and soil nutrients, carbon fractions, soil microbial diversity, and topological parameters. Under the two vegetation types of subalpine and shrubification of subalpine meadows, soil physical and chemical properties and carbon fraction indicators such as pH, TC, TN, TP, C/N, C/P, N/P, DOC, MBC, and MAOC; fungal ACE, Chao 1, Shannon, and Simpson indices; bacterial ACE, Chao, Shannon, and Simpson indices; and other microbial diversity indicators, as well as microbial network structure indicators, such as fungal positive connectivity ratio, average degree, weighted degree centrality, fungal modularity, and bacterial positive connectivity ratio, average degree, weighted degree centrality, and modularity, were all associated with soil organic carbon mineralization at different temperatures (15°C, 25°C, and 35°C) and with Q_10_ in the temperature intervals of 15°C–25°C and 25°C–35°C, which had significant positive or negative correlations, and the correlations were significant to varying degrees.

**FIGURE 9:**
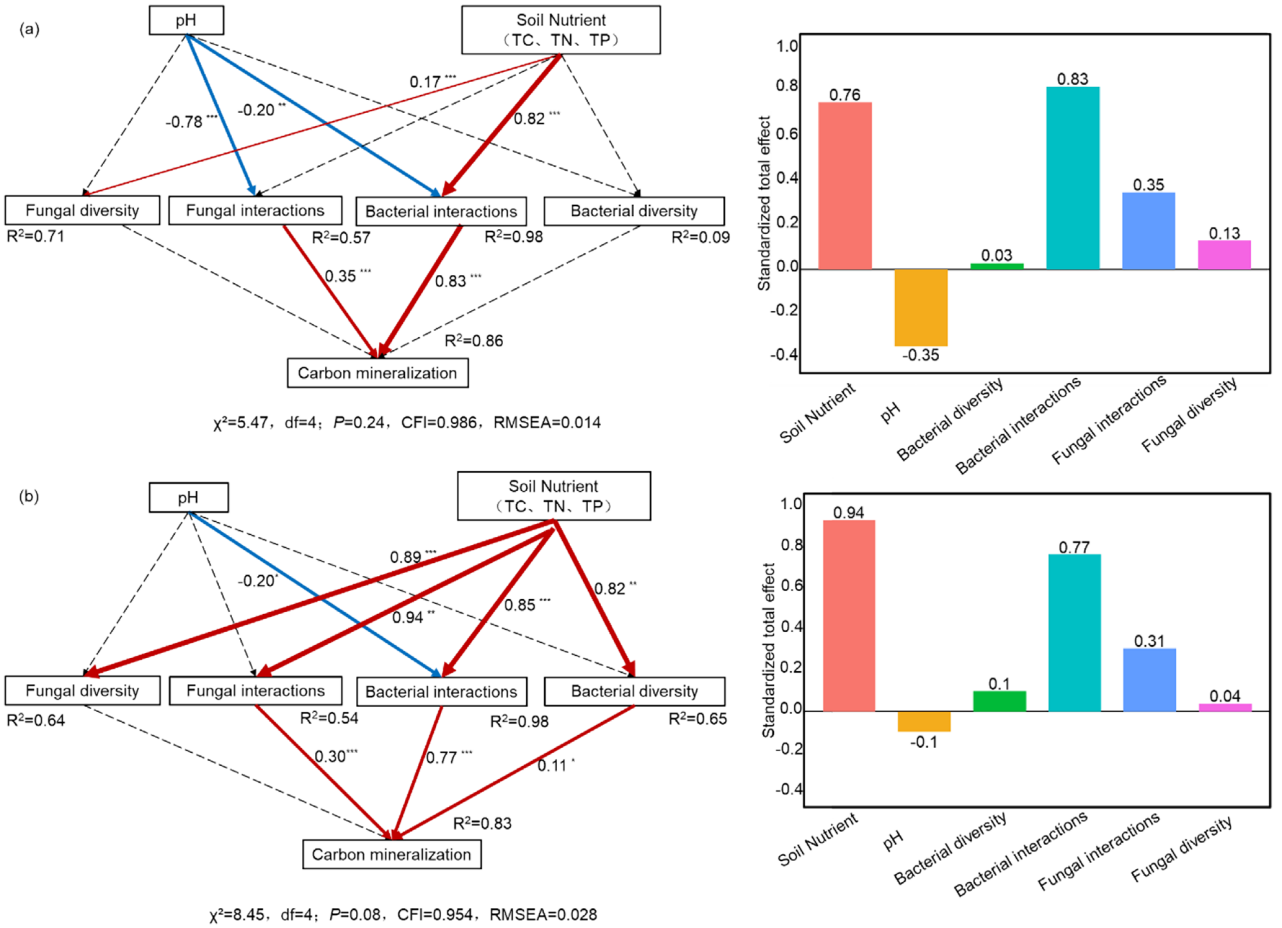
Pathway analysis diagrams of soil cumulative mineralization for various factors in native subalpine meadow (a) and shrubification of subalpine meadow (b). Soil nutrients include variables TC, TN, and TP. Fungal diversity contained variables of Sobs, ACE, Chao, Shannon, Simpson, Shannoneven, Simpsoneven, and Coverage. Bacterial diversity contained variables of Sobs, ACE, Chao, Shannon, Simpson, Shannoneven, Simpsoneven, and Coverage. Fungal interactions contained variables of Node, Number of positive edges, Average degree, Weighted degree, Modularity, and Clustering Coefficient. Bacterial interactions contained variables of Node, Number of positive edges, Average degree, Weighted degree, Modularity, and Clustering Coefficient. Carbon mineralization includes variables the total cumulative carbon mineralization amounts under the temperatures of 15°C, 25°C, and 35°C. In the model, the direction of arrows indicates the causal relationships and action pathways between variables, with the numbers adjacent to the arrows representing the load factors among variables. Red lines signify positive effects, blue lines denote negative effects, and black lines indicate nonsignificant effects; simultaneously, the width of the arrows is positively correlated with the strength of the path coefficients. *R* ^2^ represents the percentage of variance explained. Dashed lines stand for paths with nonsignificant coefficients, and significance levels are marked by asterisks (***p < 0.001, **p < 0.01, *p < 0.05).

To assess the contribution of various soil variables, including soil nutrients, pH, carbon fractions, microbial diversity, and networks to soil organic carbon mineralization (Figure 9), we used pathway modeling analyses, which showed statistical significance at the *p* = 0.01 level. SEM results showed that soil pH, soil nutrients, and microbial diversity and networks exhibited strong correlations with SOC mineralization in both subalpine and shrubification of subalpine meadow soils. Soil microorganisms played a particularly important role in carbon mineralization in meadow soils, influencing SOC mineralization in addition to directly contributing to carbon input. The contribution of bacterial diversity to carbon mineralization was significantly enhanced in shrubification of subalpine meadows, where soil bacterial diversity had a significant positive effect on carbon mineralization, while the effect was weakened in native subalpine meadows. Meanwhile, soil nutrients and pH acted as the core driving variables, and regulated the microbial community characteristics such as bacterial and fungal diversity, network complexity and other characteristics of microorganisms to synergize the mineralization, with the strongest total effect of soil nutrients, the key mediator of bacterial network, and pH through the regulation of bacterial diversity, network complexity and other microbial characteristics. networks as key mediators, and pH acting indirectly through the microbial community. In native subalpine meadows, the soil‐microbe‐mineralization mechanism maintained the intrinsic pattern; in shrubification of subalpine meadows, the mechanism was reshaped, and the contribution of fungi to mineralization increased in weight. The goodness of fit was 0.86 (*χ* ^2^ = 5.47, df = 4, *p* = 0.24, CFI = 0.986, RMSEA = 0.014) for native subalpine meadows and 0.83 (*χ* ^2^ = 8.45, df = 4, *p* = 0.08, CFI = 0.954, RMSEA = 0.028) for shrubification of subalpine meadows, strongly suggesting that microorganisms, in concert with soil factors, have a significant regulatory effect on the mineralization process, and that shrubification drives the differential response of microbial communities involved in the mineralization mechanism.

## 4 Discussion

### 4.1 Shrubification Alters Soil Physicochemical Properties and Carbon Fractions

Shrubification induced distinct distribution patterns of TN, TP, and TC across soil depths compared to native subalpine meadows (Figure 1a–c). TC accumulation is primarily attributed to the apomictic carbon input pattern of shrubby vegetation, unique root exudate composition, and altered root system distribution (Montané et al. 2010). The observed TN increase is largely driven by biological nitrogen fixation associated with leguminous shrubs, while elevated TP levels result from shrub‐mediated conversion of phosphorus into less available forms, thereby reducing losses via runoff and leaching to enhance soil TP retention. These nutrients constitute not only the fundamental basis for plant growth but also serve as key limiting factors regulating soil carbon cycling through their influence on microbial activity (Wang et al. 2025).

Concurrently, shrubification altered soil pH (Figure 1f), likely through processes disrupting H^+^ production‐consumption balance: firstly, increased quantity and quality of plant residues promoting nitrification in acidifying alkaline soils; secondly, enhanced shrub uptake of base cations, reducing soil alkalinity and leading to H^+^ accumulation in root exudates to maintain charge balance. As a critical factor, soil pH indirectly modulated SOC mineralization by affecting microbial metabolic activity and the optimal conditions for SOC degrading enzymes (Jia et al. 2022). Additionally, soil stoichiometric ratios (C/N, C/P, N/P) reflected microbial access to carbon and nutrients (Mou et al. 2023). Optimal C/N ratios enhanced SOC decomposition by balancing microbial carbon and nitrogen demands, whereas elevated C/P or N/P ratios indicate nutrient limitation, suppressing microbial activity and reducing mineralization rates. Shrubification‐induced increases in TC relative to TN and elevated C/N ratios (Figure 1d) may thereby feedback‐regulate soil C, N, and P bioavailability through altered microbial community dynamics.

Shrubification altered SOC content (Figure 2a) throughout soil horizons by modifying vegetative carbon input pathways and microbial regulation of organic carbon transformation. Compared to herbaceous plants, shrubs exhibit lower nutrient conservative efficiency, producing apoplastic materials rich in decomposition‐resistant compounds (e.g., cinnamyl alcohol) that degrade more slowly (Mou et al. 2024). Concurrently, shrubification increases litter input, net primary productivity, and deep root carbon deposition, collectively promoting SOC storage (Cai et al. 2024). The SOC accumulation primarily results from decelerated SOC turnover rates under shrub cover.

DOC and MBC, as active carbon pools, are regulated differentially. DOC dynamics respond to root exudate fluxes, while MBC dynamics are governed by microbial biomass turnover and decomposition of soil organic matter (Mou et al. 2023). Shrub canopies reduce surface temperature and soil water evaporation, whereas root‐induced macropores enhance infiltration, accelerating DOC leaching to deeper horizons or groundwater and decreasing surface DOC pools (Figure 2f). In shrub‐encroached systems, rapid shrub growth competitively reduces herbaceous cover and biodiversity through light and nutrient limitation (Zhang et al. 2022). This diminishes litter input and root exudates, thereby inhibiting microbial growth and reducing MBC (Figure 2e). The resulting high C/N environment, characterized by carbon‐enriched but nitrogen‐deficient, limits microbial metabolic activity (due to nitrogen demands for biomass synthesis), slowing organic matter decomposition. Undecomposed organic carbon (especially low‐molecule‐weight precursors) readily combines with soil minerals (e.g., clays, iron‐aluminum oxides), promoting the formation of MAOC (Figure 2d). Moreover, shrubs create a fertile island effect, altering carbon storage patterns in grassland ecosystems by modifying root and soil pore structures to enhance nutrient acquisition and facilitate deep‐soil carbon storage (Gan and Hu 2025; Wang et al. 2025).

### 4.2 Shrubification Significantly Reduces SOC Mineralization and Q_10_

Soil mineralization exhibited an exponential decay pattern (Figure 4a–f), consistent with prior studies (Dong et al. 2022; Li et al. 2023). Using the decay model (Formula: 2–4), significant differences (*p <* 0.05) in SOC mineralization responses to temperature were observed between native subalpine meadow and shrubification of subalpine meadow soils across depths (Figure 4a–f). Meadow soils showed higher initial mineralization rates (*a*‐values), with temperature‐dependent differences in mineralization rates between vegetation types amplifying at elevated temperatures. This reflected the abundant substrate resources in meadow ecosystems, where warming enhanced microbial activity (Cheng et al. 2022) and stimulated soil respiration under optimal conditions (Baumert et al. 2021). Homogeneous carbon distribution and microbial colonization patterns further contributed to elevated mineralization in meadows.

The decay constant *k*‐value was significantly lower at 15°C and 35°C than at 25°C across all treatments. This likely occurred because 25°C approximated field conditions and maintained stable microbial function during incubation. Notably, *k*‐values remained consistently low in shrub soils (0.01–0.03) compared to meadows (0.02–0.04) (Figure 4a–f), primarily due to shrubification‐mediated pH regulation that indirectly suppresses mineralization rates. Soil depth exerted minimal influence on respiratory decay rates, as evidenced by nonsignificant *k*‐value differences across horizons.

Shrubification of subalpine meadows exhibited lower Q_10_ in the 0–5 cm soil depth compared to native subalpine meadows (Figure 5), with significant Q_10_ suppression in the 5–10 cm soil layer at 25°C–35°C, while no change occurred at 15°C–25°C. This reveals dual regulation of Q_10_ by temperature intervals and soil depth during shrubification, partially supporting our hypothesis. Soil C/P, N/P, and TP emerged as key drivers of Q_10_ enhancement (Jia et al. 2022). In the 5–10 cm and 10–20 cm soil layers, diminished plant‐derived inputs and limited accumulation of microbially processed organic matter under shrubification attenuated stoichiometric, substrate, and microbial diversity controls on Q_10_. Consequently, temperature sensitivity fluctuations were confined to 0–10 cm soils at 25°C–35°C due to thermal microenvironment responses. Reduced bacterial diversity and altered network topology (diminished connectivity) in the shrubification of subalpine meadows further exacerbated heterogeneous SOC decomposition regulation across soil depths and temperatures: soil microbial processed matter buffered Q_10_ changes in shallow soils, whereas substrate limitations and reduced microbial activity maintained stable sensitivity in deeper horizons (Cheng et al. 2023).

### 4.3 Bacterial Dominance in Carbon Mineralization and Functional Differentiation From Fungi During Shrubification

Consistent with our first hypothesis and findings by Wagg et al. (2019), shrubification significantly increased fungal diversity indices relative to native subalpine meadows (Figure S2b). Shrubification creates heterogeneous microhabitats through altered vegetation structure. The litter and root exudates of shrubs provide diverse nutrient sources and habitats for fungi, while increased microclimatic heterogeneity supports niche differentiation. Concurrently, shrub plants and mycorrhizal fungi symbioses promote the development of specific fungal groups and shifts in plant community composition bring new resources pattern and selective pressures, which drives fungal differentiation (Fanin et al. 2022). Cross‐domain interactions with bacteria further generate fungal niches through competition, symbiosis, and microhabitat modification (Cheng et al. 2023; Shu et al. 2023). Fungi evolving under shrubification pressure develop specialized physiological traits and metabolic strategies. These adaptations enable optimized resource acquisition in altered edaphic conditions‐particularly under asymmetric nutrient distribution and water competition—thereby enhancing abundance, richness, and diversity (Wan et al. 2023). Nutrient‐stimulated hyphal expansion occurs rapidly under shrub canopies. Fast‐growing fungi exploit elevated soil nutrients to extend hyphal networks, creating microaggregate structures that physically increase interstitial colonization space (De Oliveira et al. 2020).

In contrast, bacterial richness and diversity increased significantly only in the 0–5 cm soil layer (Figure S2a), which is consistent with the research findings of Xiang et al. (2018), who also reported surface‐layer‐specific microbial community shifts under vegetation changes. This pattern likely results from drastic alterations in the microenvironment driven by shrub encroachment. Specifically, shrub litter and root exudates tend to concentrate in the uppermost soil layer, enriching it with diverse and labile carbon–nitrogen compounds (low‐molecular‐weight organic acids, amino acids, and polysaccharides), which promote niche differentiation and microbial coexistence. A similar mechanism of substrate‐mediated diversification had been demonstrated, showing that soil nutrient hotspots derived from plant inputs drive compositional turnover in microbial communities (Delgado‐Baquerizo et al. 2016). Moreover, the dominance of dense shallow root systems in shrublands modulates local oxygen availability and soil pH, forming fine‐scale oxygen gradients that enable simultaneous survival of aerobic taxa (Actinomycetota) and facultative anaerobes (Ren et al. 2022). Additionally, the shading effect from shrub canopies buffers surface microclimates by reducing light intensity and mitigating temperature and moisture fluctuations, which helps stabilize bacterial activity and prevent environmental stress‐induced community collapse (Liu et al. 2024). Furthermore, the formation of shrub‐mycorrhizal networks contributed to microbial recruitment by releasing signaling metabolites that promote bacterial colonization and mutualistic interactions, supporting the hypothesis of plant–microbe co‐assembly (Yang et al. 2024). In contrast, below 5 cm soil depth, the influence of shrubification markedly diminishes. Substrate inputs decline, microclimatic modifications weaken, and the relative environmental uniformity results in weaker niche partitioning. This supports the idea that deeper soils act as ecological buffers, fostering more stable microbial communities that are less responsive to aboveground vegetation shifts, a pattern consistent with the depth‐dependent sensitivity to environmental drivers (Fierer et al. 2003).

Growing evidence suggests that microbial community composition, particularly microbial network characteristics, influences soil carbon mineralization (Wang, Morrissey, et al. 2021; Morriën et al. 2017). Native subalpine meadow (Table 1). soils exhibited significantly more nodes and edges in bacterial networks than shrubification of subalpine meadow, indicating larger network scale. Microbial co‐occurrence networks comprised positive and negative correlations based on interactions between nodes (Jones et al. 2018). In shrubification of subalpine meadows, bacterial networks exhibited stronger positive correlations, reaching 60.44%, attributed to their metabolic versatility, rapid reproduction, and efficient niche partitioning that stabilize community structures (Moreno‐Gámez 2022). Enhanced dispersal capacity facilitated by small cell size and high abundance, diversity further enables broad spatial occupation and complex interaction maintenance (Zheng et al. 2024).

In contrast, fungal communities in native subalpine meadows showed stronger positive correlations. Fungal communities exhibited high environmental sensitivity due to specialized resource dependencies, limited dispersal, and narrower niche requirements, making them more vulnerable to disturbance. Consequently, shrubification of subalpine meadow showed reduced fungal positive correlations.

Topological changes such as average clustering coefficient and modularity in biological co‐occurrence networks influence ecosystem efficiency and stability (Guseva et al. 2022). In this study, shrubification of subalpine meadows exhibited lower bacterial and fungal clustering coefficients, indicating reduced species segregation and higher cross‐community connectivity, and higher network modularity, suggesting enhanced stability through compartmentalized functional units.

Key species critically maintained microbial network stability in native subalpine meadows. Bacteria communities dominated by Pseudomonadota and Bacillota, and fungal communities dominated by Ascomycota and Basidiomycota, mediate essential biogeochemical functions (Figure 7). Among bacteria (Figure 7a), Pseudomonadota, as dominant key species, convert labile organic carbon into low‐molecular‐weight organic acids via heterotrophic degradation, providing bioavailable carbon substrates. Bacillota mineralize decomposition products in surface soils, driving CO_2_ efflux. Bacteroidota degrade complex polysaccharides during early litter decomposition. Chloroflexota decompose recalcitrant organic carbon under low temperature conditions. Verrucomicrobiota facilitate plant‐fungal residue turnover and enhance plants' carbon assimilation. Actinomycetota degrade lignin to maintain stable carbon degradation, and Acidobacteriota sustain slow carbon cycling in oligotrophic environments. Collectively, these taxa regulate carbon mineralization, particularly enhancing short‐term carbon fluxes (Ren et al. 2022).

Key fungal taxa (Mortierellomycota, Basidiomycota, and Ascomycota) (Figure 7b) secrete extracellular enzymes that depolymerize organic matter into bioavailable compounds, indirectly promoting carbon mineralization. In the shrub‐encroached shrubified meadows, Ascomycota dominate keystone positions through superior environmental adaptability and resource competition strategies, enabling efficient acquisition of carbon and nitrogen across heterogeneous environments (Liu et al. 2023).

Considering the complexity of soil microenvironments, multiple factors influence carbon mineralization. Shrubification significantly decreased SOC mineralization at all incubation temperatures (15°C, 25°C, and 35°C), confirming our second hypothesis (Figure 3). After 63 days, total SOC mineralization decreased by 20.1% (0–5 cm), 17.5% (5–10 cm), and 12.2% (10–20 cm) compared to native subalpine meadows. Warming significantly promoted SOC mineralization at all soil depths, though deeper soil depths showed attenuated responses due to limited organic matter and microbial activity.

Mineralization differences between shrub‐encroached and native meadows demonstrated temperature‐depth interactions: minimal divergence at 15°C, moderate increase in surface soils (0–5 cm) at 25°C, significant reduction in surface soils at 35°C, with diminishing effects in deeper layers. Linear correlations in our study (Figure 8) revealed significant relationships between cumulative mineralization and environmental factors (soil nutrients, carbon fractions), soil microbial diversity, and topological parameters. Shrub‐encroached systems exhibited: weaker pH‐mineralization correlations, stronger nutrient‐bacterial diversity linkages, enhanced positive correlations with fungal network parameters, and increased negative correlations with bacterial topological indices.

These shifts reflect altered vegetation‐soil‐microbe interactions. Soil pH may directly limit network community formation by mediating microbial richness or regulating metabolic activities (Guseva et al. 2022). Soil nutrient availability directly influences microbial composition and symbiotic patterns. Shrubification enriched metabolically economical bacterial taxa that suppressed mineralization (Shi, Delgado‐Baquerizo, et al. 2024; Shi, Deng, et al. 2024; Kurganova et al. 2019). SEM confirmed these pathways (Figure 9), demonstrating both indirect and direct effects of pH, soil nutrients, and microbial variables on mineralization. Shrubification primarily alters microbial community structure and networks by changing soil nutrients and influences soil pH, which in turn affects carbon mineralization through microbial community composition and networks. Path coefficients indicated that soil nutrients and bacteria had stronger effects on carbon mineralization than soil pH and fungi (Explicit *β*‐values in SEM results. Figure 9). This establishes bacterial communities and soil nutrients as primary regulators of carbon mineralization processes in shrub‐encroached subalpine ecosystems.

Shrubification complexly impacts microbial diversity‐carbon mineralization relationships by altering soil nutrient availability and acidity (Chen et al. 2025). Increased fungal diversity yields functional redundancy, as taxa mediating complex carbon decomposition fail to dominate. Concurrently, decomposition efficiency faces persistent constraints from enzymatic bottlenecks, bacterial competition, and plant symbiotic carbon partitioning. Although bacteria exhibit greater genetic potential for recalcitrant carbon decomposition, insufficient synergistic flora, limited labile carbon utilizer activity, and decreased oligotrophs under shrubification hinder substrate utilization efficiency via metabolic complementarity (Xiao et al. 2024).

Microbial carbon allocation shifts further enhance soil carbon stability. Fungal symbiotic carbon preferentially channels toward biomass rather than respiration. Bacterial CO_2_ release decreases due to aeration‐mediated metabolic restructuring. High fungal diversity elevates microbial residual carbon accumulation. Consequently, shrubification elevates bacterial and fungal diversity while inhibiting carbon mineralization through: reduced synergies among decomposers, inactivation of labile carbon utilizers, and weakened trace carbon utilization capacity at both bacterial and fungal levels (Shu et al. 2023; Qi et al. 2024). Critically, these mechanisms demonstrate that microbial diversity enhancement does not drive SOC mineralization rates (Wang, Bian, et al. 2021; Wang, Morrissey, et al. 2021).

Our results demonstrate that shrubification significantly alters microbial communities' structure and networks through nutrient‐mediated pathways, ultimately suppressing carbon mineralization (third hypothesis) (Figure 6) (Table 1). Key alterations include: reduced bacterial network connectivity and structural complexity, declining fungal topological parameters, and simplified interaction patterns. The observed reduction in carbon mineralization induced by shrubification can be comprehensively explained by multi‐dimensional shifts in microbial network structure. In terms of interaction relationships, bacterial networks (Pseudomonadota‐dominated) exhibited 5.21% higher positive edge proportion in shrubification of subalpine meadows versus native meadows, though functional diversity declined due to redundant synergies. For fungal networks (Ascomycota/Basidiomycota dominated), 2.77% reduced positive edges were observed with intensified competition, impairing decomposition of complex carbon substrates (inhibited ligninolytic pathways in Basidiomycota) (Shu et al. 2023; Cheng et al. 2022).

Regarding network connectivity, Actinomycetota (key lignin‐decomposing bacteria) displayed a 21.53% reduction in average degree and a 27.55% decrease in weighted degree, disrupting energy flow and metabolic pathway functionality. Although fungal networks (including Mortierellomycota) showed a 6.97% increase in average degree and 3.83% in weighted degree, nonfunctional redundant connections failed to enhance labile carbon decomposition (Chen et al. 2022).

Furthermore, changes in network modularity and clustering coefficients further clarify the mechanism. Increased bacterial modularity (by 0.10) and decreased clustering coefficients (by 0.07) fragmented functional decomposer networks dominated by Acidobacteriota, reducing intra‐module metabolic synergy. For fungi, the simultaneous occurrence of both increased modularity and decreased clustering coefficients has weakened core functions of dominant decomposers (Ascomycota, Basidiomycota), diminishing decomposition of complex carbon. These processes were driven by consequences of shrubification‐induced, including heterogeneous nutrient distribution, intensified water competition, and altered litter biochemistry (increased lignin content), collectively constraining carbon cycling efficiency: carbon cycle efficiency (Zhang et al. 2024).

## 5 Conclusions

This study demonstrates that subalpine meadow shrubification reshapes soil carbon cycling through coordinated alterations of physicochemical properties, carbon fractions, and microbial communities. Key findings revealed: (1) Shrubification acidified soils, enriched nutrients, reduced labile carbon (DOC, MBC), increased stable MAOC, and elevated surface microbial α‐diversity while reducing community network complexity. (2) SOC mineralization rates decreased exponentially with incubation time across all soil depths under shrubification, with the maximum reduction (20.1%) in 0–5 cm soil. Shrubification significantly reduced Q_10_ values in the 5–10 cm soil layer at 25°C–35°C, indicating weakened temperature sensitivity. (3) SEM delineated regulatory mechanisms: in native subalpine meadows, soil pH regulated mineralization via fungal diversity and network interactions, whereas in shrubification of subalpine meadows established nutrients driven regulation that remodeled bacterial networks (diminished Pseudomonadota‐Actinomycetota connectivity) and weakened fungal synergistic catabolism (Ascomycota, Basidiomycota). These results establish a context‐dependent framework where pH and nutrients differentially regulate SOC mineralization, with shrubification mitigating CO_2_ emissions through reduced mineralization rates and diminished temperature sensitivity under warming conditions.

## Author Contributions

**Pengli Hou:** data curation (lead), formal analysis (lead), writing – original draft (lead). **Jia Mi:** conceptualization (equal), funding acquisition (equal), methodology (equal), project administration (lead), writing – review and editing (equal). **Qianru Ren:** investigation (equal), methodology (equal). **Yao Su:** investigation (equal). **Jincheng Shi:** investigation (equal). **Jing Shi:** conceptualization (equal), funding acquisition (equal), methodology (equal), writing – review and editing (equal). **Kuanhu Dong:** methodology (equal), resources (equal). **Baofeng Chai:** methodology (equal), resources (equal).

## Funding

This project was supported by the National Natural Science Foundation of China (32271632, U22A20576), Fundamental Research Program of Shanxi Province (202203021211303, 202203021221014).

## Conflicts of Interest

The authors declare no conflicts of interest.

## Supporting information


**Figure S1:** Species composition of microbial communities at the phylum level.
**Figure S2:** Bacteria (a) and Fungi (b) diversity values in 0–5, 5–10, and 10–20 cm soil depths of subalpine and shrubification of subalpine meadows.
**Figure S3:** NMDS analysis patterns of microbial communities in subalpine and shrubification of subalpine meadows.
**Table S1:** Information on PCA dimensionality reduction.

## Data Availability

The authors confirm that the data supporting the findings of this study are available within the article and/or its Supporting Information. The raw data used in this article is only for review and can be obtained through this link https://figshare.com/s/6784c6dd1afa8d7022d2.
