# Supplementary material for: Shrub Encroachment Rewires Microbial Networks to Suppress Soil Organic Carbon Mineralization in Subalpine Meadows
Source: Ecol Evol. 2026 Jun 29;16(7):e73922. doi: 10.1002/ece3.73922 (PMC13312142; doi:10.1002/ece3.73922)
Supplement: Supplementary file 1 — Figure S1: Species composition of microbial communities at the phylum level. Figure S2: Bacteria (a) and Fungi (b) diversity values in 0–5, 5–10, and 10–20 cm soil depths of subalpine and shrubification of subalpine meadows. Figure S3: NMDS analysis patterns of microbial communities in subalpine and shrubification of subalpine meadows. Table S1: Information on PCA dimensionality reduction. [file ECE3-16-e73922-s001.docx]

**Supplementary Materials**


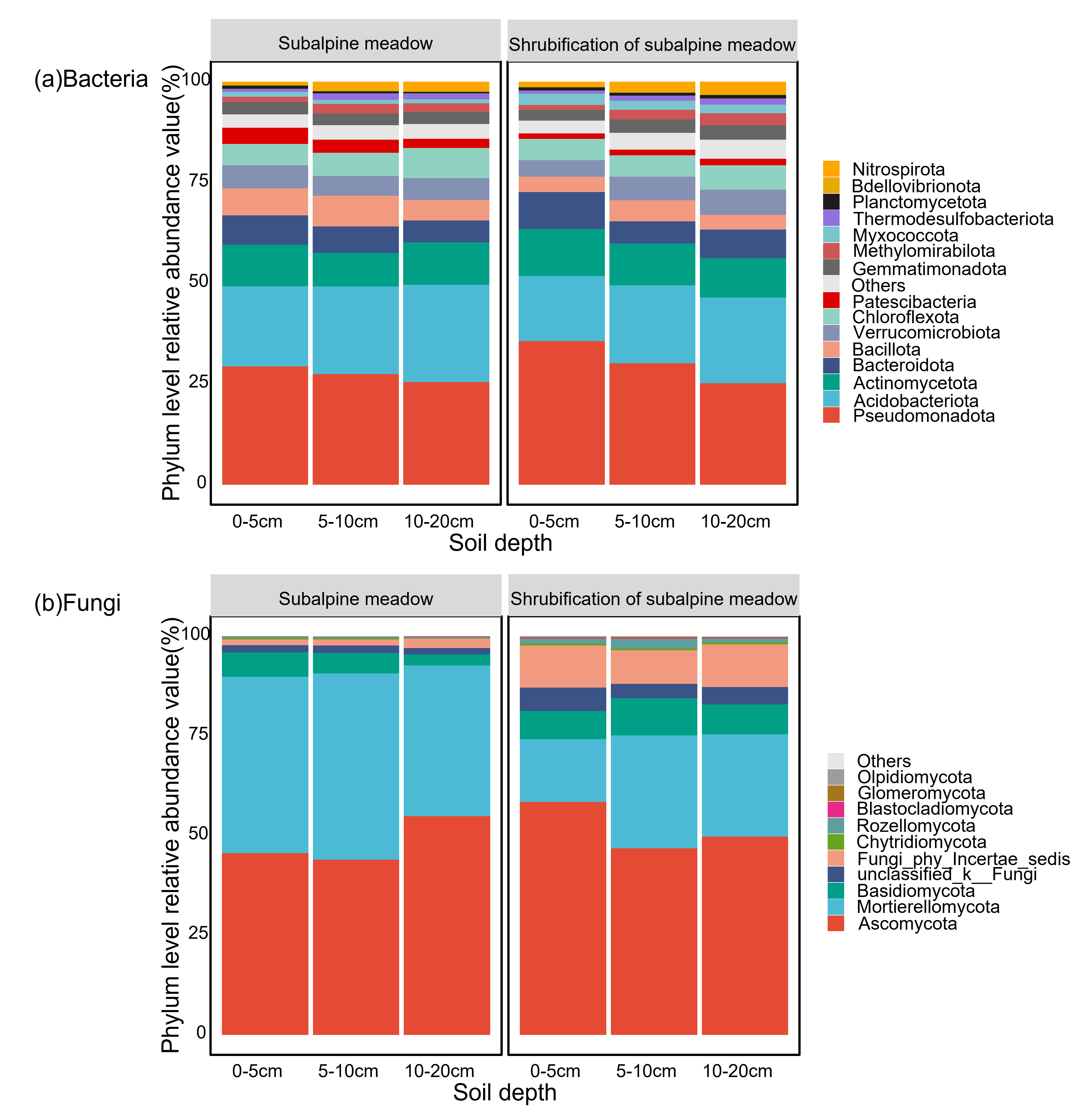


Fig. S1. Species composition of microbial communities at the phylum level.


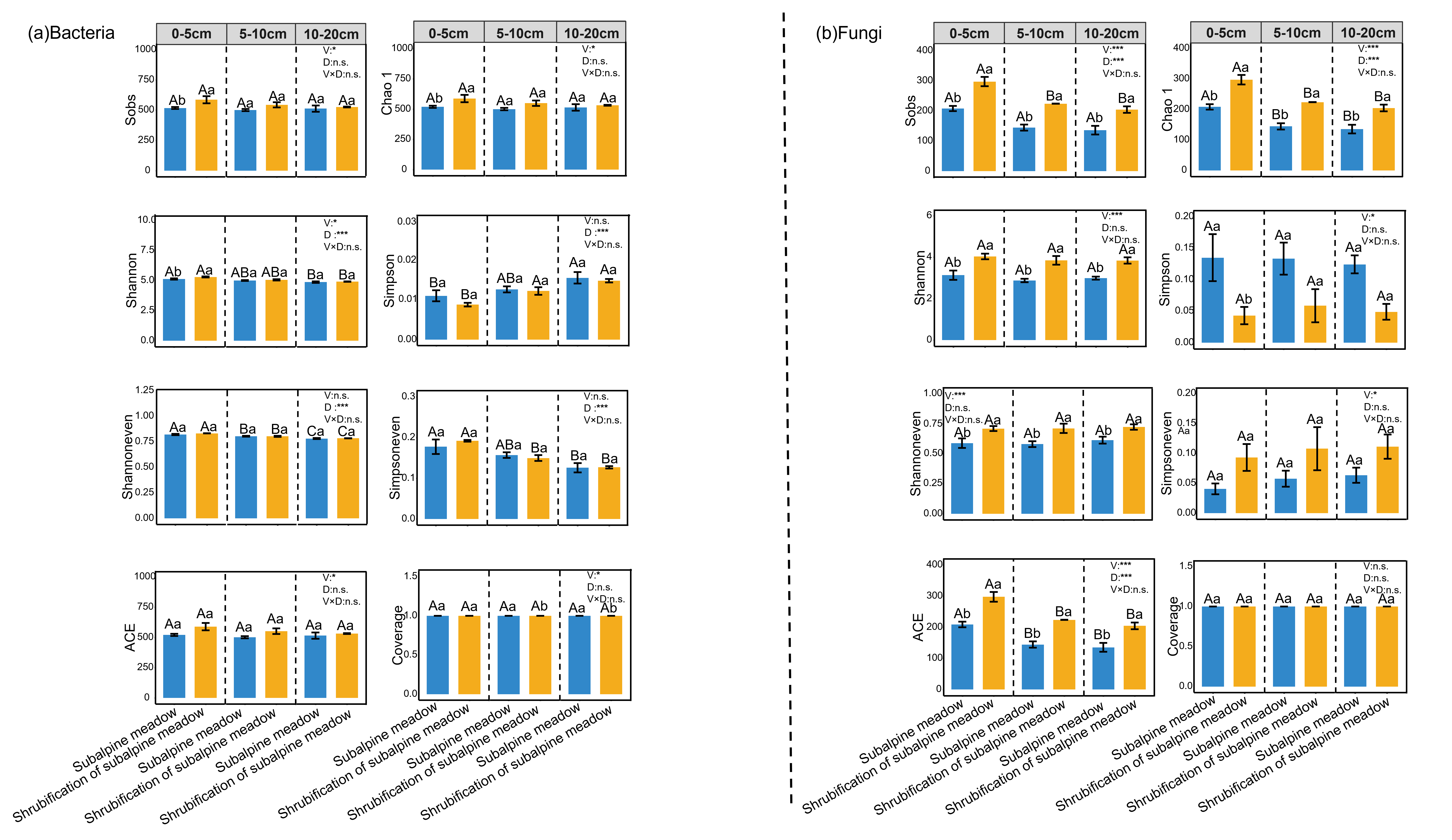


Fig. S2. Bacteria (a) and Fungi (b) diversity values in 0-5 cm, 5-10 cm, and 10-20 cm soil depths of subalpine and shrubification of subalpine meadows.


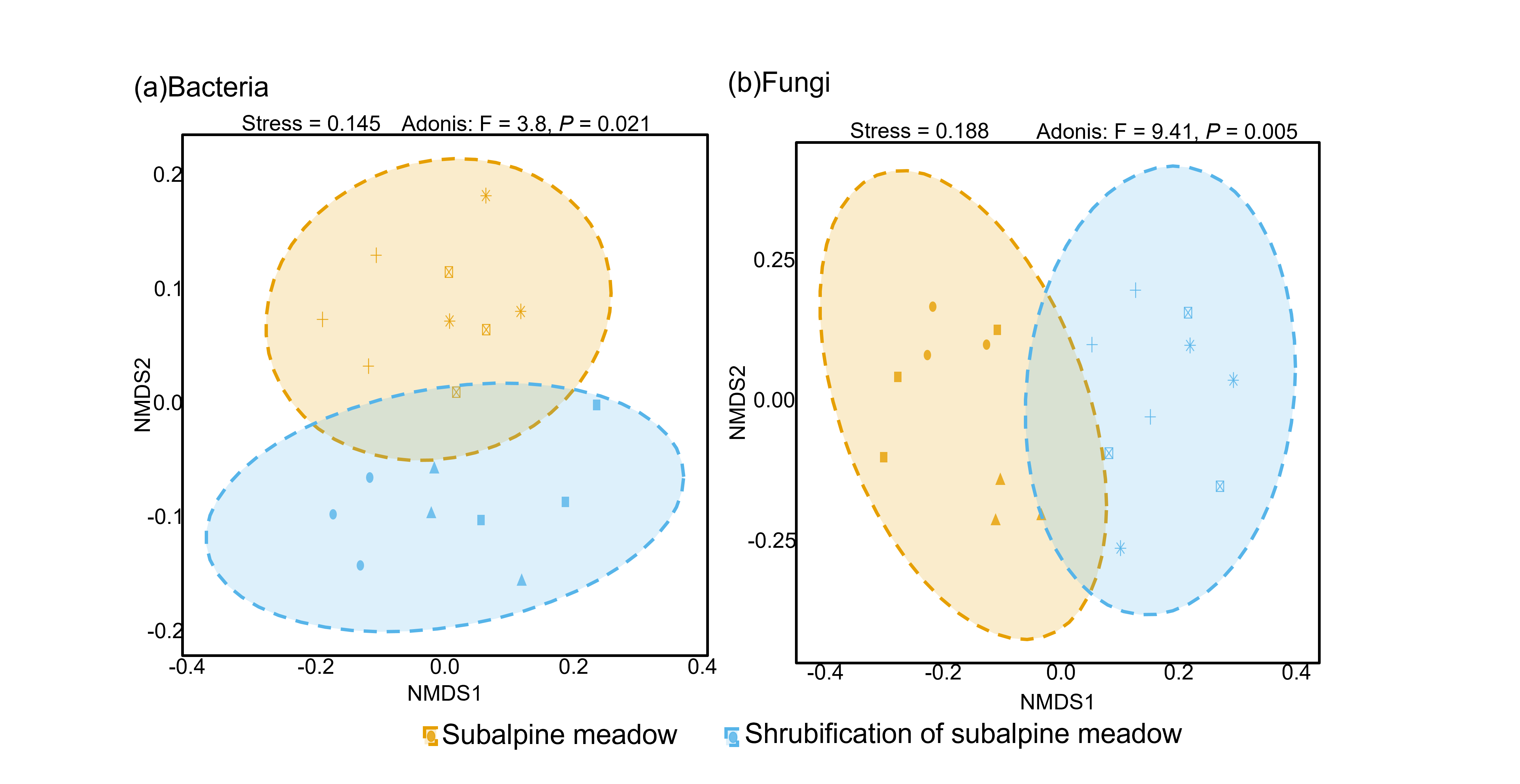


Fig. S3. NMDS analysis patterns of microbial communities in subalpine and shrubification of subalpine meadows.

Table S1 Information on PCA dimensionality reduction.

| Meadow type | Parameters | Indicators | PC1(%) |
| --- | --- | --- | --- |
| Subalpine meadow | Soil Nutrient | TC, TN, TP | 94.6 |
|  | Fungal diversity | Sobs, ACE, Chao 1, Shannon, Simpson, Shannoneven, Simpsoneven, Coverage | 59.8 |
|  | Bacterial diversity |  | 57.8 |
|  | Bacterial interactions | Edge, Node, Proportion of positive edges (%), Average degree, Weighted degree, Modularity, Average clustering coefficient | 60.8 |
|  | Fungal interactions |  | 79.9 |
|  | Carbon mineralization | cumulative carbon mineralization at 15°C, 25°C, and 35°C | 98.7 |
| Shrubification of subalpine meadow | Soil Nutrient | TC, TN, TP | 94.1 |
|  | Fungal diversity | Sobs, ACE, Chao 1, Shannon, Simpson, Shannoneven, Simpsoneven, Coverage | 50.5 |
|  | Bacterial diversity |  | 77.8 |
|  | Bacterial interactions | Edge, Node, Proportion of positive edges (%), Average degree, Weighted degree, Modularity, Average clustering coefficient | 86.0 |
|  | Fungal interactions |  | 78.7 |
|  | Carbon mineralization | cumulative carbon mineralization at 15°C, 25°C, and 35°C | 99.8 |
